# Cancer patients' concerns regarding access to cancer care: perceived impact of waiting times along the diagnosis and treatment journey

**DOI:** 10.1111/j.1365-2354.2011.01311.x

**Published:** 2012-05

**Authors:** C Paul, M Carey, A Anderson, L Mackenzie, R Sanson-Fisher, R Courtney, T Clinton-Mcharg

**Affiliations:** The University of Newcastle, Health Behaviour Research Group and Priority Research Centre for Health Behaviour (PRCHB), Hunter Medical Research Institute (HMRI)Callaghan, NSW, Australia

**Keywords:** cancer patients, health services accessibility, psychosocial aspects, diagnosis, treatment, vulnerable populations

## Abstract

Waiting times can raise significant concern for cancer patients. This study examined cancer patients' concern levels at each phase of waiting. Demographic, disease and psychosocial characteristics associated with concern at each phase were also assessed. 146 consenting outpatients (*n*= 146) were recruited from two hospitals in Sydney, Australia. Each completed a touch-screen computer survey, asking them to recall concern experienced regarding waiting times at each treatment phase. Approximately half (52%) reported experiencing concern during at least one treatment phase, while 8.9% reported experiencing concern at every phase. Higher proportions of patients reported concern about waiting times from: deciding to have radiotherapy to commencement of radiotherapy (31%); the first specialist appointment to receiving a cancer diagnosis (28%); and deciding to have chemotherapy to commencement of chemotherapy (28%). Patient groups more likely to report concern were those of lower socio-economic status, born outside Australia, or of younger age. Although a small proportion of patients reported very high levels of concern regarding waiting times, the experience of some concern was prevalent. Opportunities for reducing this concern are discussed. Vulnerable groups, such as younger and socio-economically disadvantaged patients, should be the focus of efforts to reduce waiting times and patient concern levels.

## INTRODUCTION

### The importance of access to high-quality cancer care

Cancer is an international health priority and a major cause of morbidity and mortality worldwide (World Health Organisation 2009). Accordingly, the identification of key indicators of high-quality cancer care has received much attention (Organisation for Economic Development and Co-operation 2009; Institute of Medicine 2011). Access to care is a key indicator of quality care, as indicated by its inclusion in key documents assessing care standards (The Royal College of Physicians & The Royal College of Radiologists 1993; New South Wales Health 2003; Health Canada 2004; Agency for Healthcare Research and Quality 2009) and population health monitoring (Andersen 2008). Access to care involves not just the availability of a service, but also the ability to utilise that care (Aday & Andersen 1974). The receipt of timely attention is central to high-quality care in that delays in receiving care may lead to more advanced disease (Mohammed *et al*., 2011) and subsequently reduced length of life (Richards *et al*. 1999; Fahmy *et al*. 2006; Teppo & Alho 2009).

### Timely access to cancer care as a measure of quality

A number of authors have explored delays in the processes of cancer care from the first experience of a symptom to the receipt of treatments (Salomaa *et al*. 2005; Evans *et al*. 2007; Olesen *et al*. 2009). These explorations provide a useful framework for conceptualising the patient experience as a series of ‘waiting times’ between crucial treatment phases. Olesen *et al*. (2009) identified these crucial phases as the time between the:

1. First contact with a primary care provider and initiation of symptom investigation;
2. Initiation of symptom investigation and subsequent referral;
3. Hospital/specialist referral and first hospital/specialist visit; and
4. Referral for treatment and the commencement of treatment.

Internationally, guidelines or standards in relation to acceptable waiting times for these crucial phases of cancer care vary (The Royal College of Physicians & The Royal College of Radiologists 1993; Department of Health 2000; Manpower and Standards of Care in Radiation Oncology Committee 2000; New South Wales Health 2003). Such guidelines are generally focussed on maximising a patient's length of life. However, there is a growing emphasis on the need to minimise psychosocial impacts which may be caused by delays in care (Department of Health 2000; Jones *et al*. 2001; New South Wales Health 2003; Cancer Care Ontario 2008).

The literature has typically focussed on waiting times (delays) which can have a direct impact on disease outcome. Regardless of whether there is medical risk associated with a delay in accessing care, such delays may have an important psychosocial impact on the patient and his or her family. The consumer experience is an important element for assessing the impact of structures and processes in the care pathway (Sanson-Fisher *et al*. 2009) given that these elements are often not observable to consumers. It has been argued that endpoint measures, such as patient satisfaction with care, represent an external validation of realised access to care (Aday & Andersen 1974). Previous studies have focussed on patient satisfaction (Cancer Institute New South Wales 2009) and actual waiting times (Gorey *et al*. 2009; Bilimoria *et al*. 2011) without gaining a clear sense of the level of patient concern which arises as a result of the perception of waiting.

A relatively new approach to assessing the impact of waiting times on patients is to assess the level of concern arising at critical phases of care. Patient concern regarding waiting times may represent a combination of: (1) the actual or perceived medical risk associated with a delay; (2) patient expectations of care and treatment; and (3) the quality of communication about the waiting times provided by health professionals. Despite reported variations in acceptable waiting times for care, relatively little attention has been directed towards the patients' level of concern in relation to the experience of waiting. Studies which have focussed on the patient experience of waiting times have primarily measured patient satisfaction rather than concern (Gesell & Gregory 2004; Absolom *et al*. 2006; Groff *et al*. 2008).

### Factors potentially associated with patient experiences of timely access to care

Models which conceptualise access to care from the patient's perspective have identified a range of factors which may be related to actual utilisation of health services, including patient attitudes, socio-demographic characteristics, and structural aspects of treatment centres (Andersen 1995). A number of factors have also been associated with delays in access to cancer care, including greater geographical distance from care (Sowden *et al*. 1997; Campbell *et al*. 1999; Jones *et al*. 2008; Onega *et al*. 2008; Drury & Inma 2010), income (van Doorslaer *et al*. 2006), ethnicity (Shi & Stevens 2005) and health insurance (Hoffman & Paradise 2008). Therefore, these factors might also be associated with greater levels of patient concern about such delays.

While the evidence regarding the effect of socio-demographic factors, such as increased distance to care, on disease outcomes is mixed (Sowden *et al*. 1997; Campbell *et al*. 1999), equity of patient access is considered an integral part of providing high-quality care (Institute of Medicine 2001). Therefore, an exploration of patient concerns regarding waiting times for treatment and care should also explore the role that socio-demographic factors may play in experiencing such concerns.

### Aims

Among cancer patients attending outpatient radiation therapy appointments, this study aimed to identify:

(i) The proportion of patients reporting any level of concern regarding the time elapsed between each of:
  - First symptom-related visit to the General Practitioner (GP), and referral to a cancer specialist,
  - Referral to a cancer specialist, and first appointment with the cancer specialist,
  - First appointment with the cancer specialist and receiving a cancer diagnosis,
  - Decision to have surgery and the date of surgery,
  - Decision to have radiotherapy and commencement of radiotherapy,
  - Decision to have chemotherapy and the commencement of chemotherapy;
    The proportion of patients reporting ‘moderate or high’ levels of concern regarding waiting times at the above phases of treatment.
The proportion of patients reporting ‘any’ level of concern for multiple phases of treatment.
Associations between demographic characteristics, disease characteristics, and self-reported psychological distress, and reporting any level of concern at each phase of treatment.

## METHODS

### Design and ethical approval

A cross-sectional, self-report survey regarding cancer care experiences was completed by participants via touch-screen computer. Ethical approval for the study was obtained from the New South Wales (NSW) Population and Health Services Research Ethics Committee and the University of Newcastle Human Research Ethics Committee. Relevant institutional ethics approvals were also obtained.

### Sample

Participants were cancer outpatients recruited from radiation therapy treatment units at two hospitals in Sydney, Australia between March and September 2010. Eligible patients were: aged 18 years or older; diagnosed with any type of cancer; and sufficient in English to complete the survey. Patients who were attending the clinic for the first time were excluded, as at least one prior visit was considered necessary in order to answer a number of the items in the wider cancer survey. Patients who were judged by staff as physically or mentally incapable of completing the survey were also excluded.

### Procedure

A nurse from each clinic identified eligible patients from daily clinic appointment lists. Patients were then approached by a research assistant (RA) while waiting for their appointment. Consenting participants were asked to complete a survey using a touch-screen computer. The RA explained the survey content and navigation and logged participants onto the survey using a unique ID code. Participants were given the option of resuming the survey after treatment if they were unable to complete it prior to their appointment.

### Measures

Previous research (Salomaa *et al*. 2005; Olesen *et al*. 2009), best practice guidelines (New South Wales Health 2003) and consultations with oncologists were used to identify significant treatment phases throughout the cancer journey where waiting times may occur. Survey items were revised following pilot testing with 66 cancer outpatients attending radiation clinics over a 2-week period in February 2010. The patient survey was programmed into a Dell touch-screen computer using Digivey survey software. The following modules were embedded within the larger survey.

#### Level of concern regarding waiting times at each treatment phase

Six items addressing the six treatment phases (outlined in the aims above) were presented, with respondents indicating their recalled level of concern regarding waiting times for each phase.

For example: ‘The length of time I waited between my doctor deciding I was ready for surgery and having surgery to remove the cancer was …’– Not at all concerning; Slightly concerning; Moderately concerning; or Very concerning. Each item response scale also included a ‘not applicable’ option such as ‘Have not had surgery to remove the cancer’.

#### Demographic and disease characteristics

Data on: age; gender; postcode; country of birth; health insurance status; living arrangements; cancer diagnosis; cancer recurrence; time since diagnosis; treatment aim; number of outpatient appointments; and number of oncology appointments, were also collected via patient self report. Socio-economic status (SES) was categorised as low, medium or high based on the Socio-economic Index for Areas (SEIFA) (Australian Bureau of Statistics 2006). Geographical location was categorised as urban, regional and rural and was determined by postcode using the Accessibility/Remoteness Index of Australia (ARIA+) (Trewin 2006).

#### Levels of clinical distress

The Hospital Anxiety and Depression Scale (HADS) (Zigmond & Snaith 1983) was used as a psychometrically rigorous measure of clinically significant distress at the time of the survey. Caseness for anxiety or depression was defined as a score of eight or above on the corresponding subscale of the HADS (Love *et al*. 2002). HADS subscale scores were included in statistical models as variables which could be potentially associated with levels of reported concern (Moorey *et al*. 1991; Lloyd-Williams 2001; Keller *et al*. 2004; Walker *et al*. 2007).

### Statistical analysis

Frequencies, proportions and 95% confidence intervals (CI) were used to describe the demographic characteristics of participants and their levels of concern regarding waiting times, both overall and at each relevant phase of treatment. Chi-square and logistic regression analyses were used to determine associations between patients': demographic characteristics, disease characteristics and levels of psychological distress, and experiencing concern for each phase of treatment. The following demographic, disease, and distress characteristics were included in the chi-square analysis: gender; SES (SEIFA); geographical location (ARIA+); health insurance; living arrangements; country of birth; treatment aim; time since diagnosis; cancer recurrence; anxiety; and depression. For each phase of treatment, variables with a value of *P* < 0.25 on the chi-square test were retained for inclusion in the logistic regression. Patient age in years was also added to all logistic regressions as a continuous variable. Variables with a value of *P* < 0.10 in the logistic regression were removed, and regressions were rerun with the remaining variables to identify significant associations.

## RESULTS

### Response rate and demographic characteristics

Of the 246 eligible patients, 218 consented to take part, giving a consent rate of 89%. Of the 218 patients who began answering the questionnaire, 146 completed it giving a completion rate of 67%. Non-completion was primarily due to appointment waiting times being shorter than expected.

The demographic and disease characteristics of participants are presented in Table 1. The age of participants ranged from 19 to 90 years, with a mean age of 60 (SD = 14.1 years). Almost 70% of patients reported they were unsure whether or not they had had a recurrence of their cancer or a secondary cancer diagnosis. One-fifth of patients reported that they lived alone. There were no patients from rural areas, as defined by ARIA+ (Trewin 2006).

**Table 1:** Participant demographic and disease characteristics (*n*= 146)

Patient demographics	*n* (%)	95% CI
Gender		
Male	70 (48)	40–56
Female	76 (52)	44–60
Cancer type		
Breast	50 (34)	27–42
Prostate	25 (17)	12–24
Head and neck	13 (8.9)	5.2–15
Brain	9 (6.2)	3.2–12
Colorectal/bowel	9 (6.2)	3.2–12
Other	40 (27)	21–35
Time since diagnosis		
≤2 years	122 (84)	77–89
>2 years	24 (16)	11–23
Second cancer diagnosis or recurrence		
Yes	38 (26)	19–34
No/not sure	108 (74)	66–81
Stage of treatment		
Receiving treatment	140 (96)	91–98
Finished treatment	6 (4.1)	1.8–8.9
Perceived treatment aim		
Cure	70 (50)	42–58
Prevention	58 (41)	33–50
Palliation	12 (8.6)	4.9–15
Health insurance		
Hospital and/or extras	87 (61)	53–69
No	56 (39)	31–47
Country of birth		
Australia	108 (74)	66–81
Other	38 (26)	19–34
Living arrangements		
With others	117 (80)	73–86
Alone	29 (20)	14–27
Outpatient visits to clinic		
≤10	85 (58)	50–66
>10	61 (42)	34–50
Appointments with cancer specialist		
≤4	110 (75)	68–82
>4	36 (25)	18–32
Socio-economic status*		
Low/medium	30 (21)	15–29
High	113 (79)	71–85
Geographical location†		
Metropolitan	118 (83)	75–88
Regional	25 (17)	12–25

* Socio-economic status was categorised as low (SEIFA Deciles 1–3 or <930), medium (SEIFA Deciles 4–7 or 930 to1012) or high (SEIFA Deciles 8–10 or >1012) (Australian Bureau of Statistics 2006; Linacare 2007).

† Geographical location was categorised as Metropolitan (ARIA+ Index <0.2), Regional (ARIA+ Index 0.2–5.92), or Remote (ARIA+ Index >5.92) (Trewin 2006).

### Proportion reporting concern at each time period

#### Any level of concern

As shown in Table 2, the proportion of patients reporting any level of concern (slight, moderate or very concerned) regarding waiting times varied from 23% to 31% depending on the phase of care. Phases which had the largest proportion of patients reporting concern about the time taken to access care included: (1) from the decision to have radiotherapy, to the commencement of radiotherapy (31%); (2) from the first appointment with the cancer specialist, to receiving a cancer diagnosis (28%); and (3) from the decision to have chemotherapy, to the commencement of chemotherapy (28%).

**Table 2:** Proportion who reported experiencing concerns about the time taken to access each relevant phase of cancer care from diagnosis to treatment

	No Concern	Concern	Concern by level		
			
Length of time I waited between	*n* (%, 95% CI)	*n* (%, 95% CI)	Slightly *n* (%, 95% CI)	Moderately *n* (%, 95% CI)	Very *n* (%, 95% CI)
First symptom-related visit to the GP, to referral to a cancer specialist (*n*= 121)	91 (75, 67–82)	30 (25, 18–33)	15 (12, 7.6–20)	8 (6.6, 3.3–13)	7 (5.8, 2.8–12)
Referral to a cancer specialist, to the first appointment with the cancer specialist (*n*= 133)	102 (77, 69–83)	31 (23, 17–31)	17 (13, 8.1–20)	9 (6.8, 3.5–13)	5 (3.8, 1.6–8.8)
First appointment with the cancer specialist, to receiving a cancer diagnosis (*n*= 129)	93 (72, 64–79)	36 (28, 21–36)	26 (20, 14–28)	4 (3.1, 1.2–8.1)	6 (4.7, 2.2–10)
Decision to have surgery, to the date of surgery (*n*= 111)	86 (77, 69–84)	25 (23, 16–31)	12 (11, 6.2–18)	7 (6.3, 3.0–13)	6 (5.4, 2.4–12)
Decision to have radiotherapy, to the commencement of radiotherapy (*n*= 146)	101 (69, 61–76)	45 (31, 24–39)	26 (18, 12–25)	9 (6.2, 3.2–12)	10 (6.9, 3.7–12)
Decision to have chemotherapy, to the commencement of chemotherapy (*n*= 71)	51 (72, 60–81)	20 (28, 19–40)	9 (13, 6.6–23)	4 (5.6, 2.1–14)	7 (9.9, 4.7–20)

#### Moderate or high levels of concern

Of the patients who expressed concern at each treatment phase, more than half reported they were moderately or very concerned at the following phases: from the decision to have chemotherapy, to the commencement of chemotherapy (55%); from the decision to have surgery, to the date of surgery (52%); and from the first symptom-related visit to the GP, to gaining a referral to a cancer specialist (50%). Slight levels of concern (72% of all patients concerned) were predominant for the waiting time from the first appointment with cancer specialist, to receiving a cancer diagnosis.

### Proportion reporting any level of concern at multiple phases of treatment

Over 50% of participants reported experiencing concern about waiting times at one or more phases of cancer treatment relevant to them. Within those experiencing concern, 17% of individuals (8.9% of all respondents, 95% CI = 5.2–15) reported experiencing concern regarding waiting times at every phase of cancer treatment relevant to them. Forty-three per cent of all respondents (95% CI = 35–51) reported concern at some of the phases of treatment relevant to them, while 48% (95% CI = 40–56) reported concern at none of the phases of treatment they had experienced.

### Associations between some level of concern and demographic, disease, and distress characteristics for each phase of treatment

#### First symptom-related visit to the GP, to referral to a cancer specialist

Four variables had a value of *P* < 0.25 on the chi-square test: time since diagnosis, *P*= 0.17; cancer recurrence, *P*= 0.18; country of birth, *P*= 0.06; and anxiety, *P*= 0.16. However, following logistic regression analysis, no significant association between these variables and concern at this phase of treatment was found.

#### Referral to a cancer specialist, to the first appointment with the cancer specialist

No variables had a value of *P* < 0.25 after performing the chi-square test indicating no association between concern at this phase of treatment and patients' demographic, disease or distress characteristics.

#### First appointment with the cancer specialist, to receiving a cancer diagnosis

Two variables (cancer recurrence, *P*= 0.16 and country of birth, *P*= 0.02) had values of *P* < 0.25 on the chi-square test. While neither of these variables showed a significant association with concern, the logistic regression analysis did reveal a significant association between patient age and concern. For younger patients, the odds of reporting concern about waiting times from the first appointment with the cancer specialist, to receiving a cancer diagnosis was increased (OR = 1.03, SE = 0.02, *P*= 0.04, 95% CI 1.00–1.06).

#### Decision to have surgery, to the date of surgery

Following chi-square analysis, five variables had values of *P* < 0.25 including: SES, *P*≤ 0.01; geographical location, *P*= 0.07; time since diagnosis, *P*= 0.14; cancer recurrence, *P*= 0.13; and country of birth, *P*= 0.07. Logistic regression analysis showed that, compared with patients from high socio-economic backgrounds, patients with low to medium SES had almost six times higher odds of reporting concern about waiting times from the decision to have surgery, to the date of surgery (OR = 5.94, SE = 3.44, *P* < 0.01, 95% CI 1.91–18.46). The odds of reporting concern at this phase of treatment were also three times higher for patients who were not born in Australia, compared with those who were (OR = 3.06, SE = 1.61, *P*= 0.03, 95% CI 1.09–8.57).

#### Decision to have radiotherapy, to the commencement of radiotherapy

While gender (*P*= 0.11), health insurance (*P*= 0.23), and living arrangement (*P*= 0.17) variables were added to the logistic regression analysis, no associations for reporting concern at this treatment phase were found.

#### Decision to have chemotherapy, to the commencement of chemotherapy

Similarly, country of birth (*P*= 0.05) was added to the logistic regression model for this phase of treatment, and although there was a strong association between country of birth and concern, it was not significant at the *P* < 0.05 level.

## DISCUSSION

### Concern about waiting times was reported across all phases of treatment

This study is unique in focussing on level of patient concern associated with perceived waiting times for diagnosis or treatment phases. One-fifth of radiation oncology outpatients who participated reported experiencing substantial levels of concern about the time which elapsed between the treatment phases examined. Levels of concern were considered to be moderate to high for about half of those who reported concern. Waiting time related to receiving a cancer diagnosis was the only phase where slight levels of concern were predominantly reported. This is surprising given that previous studies have reported high rates of anxiety while waiting for test results to confirm a cancer diagnosis (Poole 1997; Drageset *et al*. 2010). There has been considerable attention to communication skills training for oncology professionals to assist in the delivery of bad news (Ellis & Tattersall 1999; Back *et al*. 2005). Therefore, this finding may reflect that health professionals have greater skills and awareness of the need to provide appropriate reassurance to patients waiting for the results of diagnostic tests than for other phases in the care trajectory.

Patient concern may relate to expectations that any delay will reduce the chances of a positive treatment outcome, along with anxiety regarding expected risks and side effects of treatment. As cancer diagnosis and treatment pathways are often lengthy, multi-staged, physically difficult and uncertain in outcome (Fitch *et al*. 2003; Clark & Talcott 2006), some level of patient concern may be unavoidable. This might be considered a strong imperative for attempting to minimise any avoidable distress for this population.

### Concern about waiting times was relatively widespread across participants

Close to half of the sample reported concern at some phase of the cancer diagnosis and treatment pathway, with 8.9% reporting concern at every phase. Therefore, it appears unlikely that concern about waiting times is confined to a subgroup of individuals who are consistently bothered by waiting times. Further, individuals who were categorised as possible or probable cases of anxiety or depression (according to the HADS), were no more likely to report concern about waiting times than those falling below the ‘caseness’ threshold. Consistent with previous findings, it appears likely that factors associated with the experience of waiting (uncertainty combined with the actual length of time) may be the primary drivers of concern, rather than factors intrinsic to the patient or the disease type (Fitch *et al*. 2003; Sanmartin *et al*. 2007).

#### Which groups appeared to be particularly vulnerable?

Contrary to our expectations, relatively few associations were identified between disease or socio-demographic factors and levels of concern. Younger patients had slightly higher odds of reporting concern about waiting times from their first appointment with the cancer specialist to receiving a cancer diagnosis. It may be that younger adults are particularly concerned by delays during the diagnostic pathway. Compared with high SES patients, patients of low to medium SES had almost six times higher odds of reporting concern about the time taken to access surgery. The odds of reporting concern about the time taken to access surgery were also three times higher for patients who were born outside Australia, compared with Australian-born patients. Cancer patients have reported a fear of the cancer spreading during the time between diagnosis and surgery (Fitch *et al*. 2003) and high levels of psychological distress and uncertainty preoperatively (Drageset *et al*. 2010). Living in a disadvantaged area or being born in another country may present particular difficulties for arranging timely admission for surgery (Thomas *et al*. 2009). Some variations by SES in median waiting times for elective surgery in Australia have been reported (Australian Institute of Health and Welfare 2010), and migrant groups diagnosed with cancer have been reported to have poorer outcomes than non-migrants (Gotay *et al*. 2002). These findings suggest the need to explore the source of concerns about waiting times in these groups. Regardless of whether these groups actually wait longer for surgery, there is a need for additional communication, assistance or support to help manage their particular needs during the waiting time leading up to surgery (Coates 1999; Fitch *et al*. 2003). Inclusion of non-English speaking patients was beyond the scope of the present study. However, given the elevated levels of concern among English speaking patients born outside of Australia, it seems that exploration of the concerns and experiences of non-English speaking patients may be an important area for future investigation. Studies of the concerns of more culturally and linguistically diverse samples which permit a comparison of English and non-English speaking patients levels of concern may be helpful to explore this further (Gotay *et al*. 2002).

#### How might concerns be addressed or minimised?

One of the primary paths to reducing the concern over waiting times is improvements in referral patterns and treatment booking systems to minimise actual waiting times. For example, NSW Health (New South Wales Department of Health 2011) has waiting time information which is accessible online and by telephone similar to that used in the UK and Canada (Cancer Care Ontario 2008; National Health Service 2011), to assist referring doctors and their patients in choosing the most suitable treatment centre for surgery. Improving information and support options throughout the treatment trajectory is also likely to be important, not least in terms of informing patients at each step how long the wait could or should be, whether the wait is or is not likely to have an impact on their treatment outcome, and avenues for accessing information for any concerns or queries they may have while waiting.

### Limitations

The study methodology has a number of limitations including a relatively small sample of patients who were receiving radiotherapy at one of two public hospitals in metropolitan NSW Australia. This approach provided limited power to identify associations and to generalise to broader patient groups. It should also be noted that SES was assessed as an ecological (area-based) measure rather than a more direct assessment of individual-based markers such as household income (Taylor *et al*. 2001).

The survey also required patients to report on their level of concern retrospectively. Therefore, it is possible that recall bias or the outcomes of treatment may have affected patient evaluation of their level of concern at the earliest phases of diagnosis. It should also be noted that reported level of concern about waiting time may not be directly related to actual time elapsed, which was not recorded for this study.

## CONCLUSION

While it is not yet known whether longer waiting times at different phases of the illness trajectory are associated with poorer clinical or psychosocial outcomes, cancer outpatients express concerns associated with waiting times across almost every care phase from pre-diagnosis to treatment. Patient self-reported concern about waiting times provides an endpoint assessment of an important aspect of quality of care. Further investigations of the factors which underlie these concerns are warranted to understand and intervene in a manner which minimises distress to this very vulnerable patient group.
